# The effect of the interaction between fall-related self-efficacy and gait function on the occurrence of falls in community-dwelling older people

**DOI:** 10.1007/s40520-021-01807-0

**Published:** 2021-02-24

**Authors:** Naoto Kamide, Haruhiko Sato, Miki Sakamoto, Yoshitaka Shiba

**Affiliations:** 1 School of Allied Health Sciences, Kitasato University, 1-15-1 Kitasato, Minami-ku, Sagamihara, Kanagawa 252-0373 Japan; 2 Graduate School of Medical Sciences, Kitasato University, Sagamihara, Japan

**Keywords:** Aged, Fall-related self-efficacy, Falls, Gait, Japanese

## Abstract

**Background:**

Fall-related self-efficacy and gait function are known to be associated. However, whether the interaction between fall-related self-efficacy and gait function affects future falls has not been investigated.

**Aim:**

The aim of this study was to investigate the effect of the interaction between fall-related self-efficacy and spatiotemporal gait parameters on the occurrence of falls in community-dwelling older people.

**Methods:**

A total of 265 elderly persons (age ≥ 65 years) living independently in the community were recruited. For gait function, spatiotemporal gait parameters at usual and maximum effort paces were measured using a 2.4-m walkway system with embedded pressure sensors. Furthermore, changes in gait parameters between usual and maximum paces were calculated (Δgait parameters). Fall-related self-efficacy was assessed using the short version of the Falls Efficacy Scale International (Short FES-I). The occurrence of falls was prospectively investigated 6 months later. The effect of the interaction between short FES-I and gait parameters on falls was analyzed using logistic regression analysis adjusted for confounding factors.

**Results:**

Several gait parameters were significantly different by self-efficacy level. As for the effect of the interaction of fall-related self-efficacy and gait parameters on falls, smaller Δgait parameters in those with high efficacy were associated with higher odds ratios of falls, whereas Δgait parameters in those with low efficacy were not associated with falls.

**Discussion and conclusions:**

The interaction between fall-related self-efficacy and gait function appeared to affect future falls. Assessments combining fall-related self-efficacy and gait function may improve the accuracy of prediction of future falls.

## Introduction

Falling is a well-known, representative adverse health event in older people; thus, fall prevention is an indispensable issue for healthcare providers [1]. To prevent falls in older people, identification of fall risk is necessary; especially, with respect to intrinsic factors, assessing physical and psychological risk factors is important. With respect to physical aspects related to fall risk, gait function, such as gait speed, is a representative predictor of falls [2–4]. On the other hand, with respect to psychological aspects, fear of falling, or fall-related self-efficacy is known as a representative predictor of falls [5–8]. The terminology with respect to fear of falling and fall-related self-efficacy is confusing in relevant studies, and both terms tend to be used in almost the same context. However, both terms need to be distinguished according to the psychometric properties of the scale used in a particular study [5]. In the present study, fall-related self-efficacy was used, taking into account the psychometric properties of the scale used in this study.

As described above, gait function and fall-related self-efficacy were found to be predictors of falls. However, with respect to the prediction of falls in older people, it has been suggested that an assessment of fall risk by stand-alone measurement of gait speed did not have sufficient sensitivity to discriminate between fallers and non-fallers [9]. In fact, the mean difference in gait speed between fallers and non-fallers was reported to be 4 cm/s in a meta-analysis including only prospective studies [9]. However, this mean difference was smaller than the measurement error, which was reported to be 10.8 cm/s in a previous study of gait speed [10]. Similarly, it was also suggested that the sensitivity for detecting falls of the scale evaluating fall-related self-efficacy was not sufficient, having been previously reported to be 60% in a prospective study [8]. That is, a fall risk assessment for older people needs to consist of multiple components such as physical and psychological factors. Therefore, we hypothesized that an assessment incorporating both gait function and fall-related self-efficacy could improve accuracy to predict falls more than an assessment using respective stand-alone measurements.

Several studies have reported that physical functions and fall-related self-efficacy are associated with each other [11]. In particular, it has been reported that fall-related self-efficacy is associated with gait speed independently of physical and cognitive functions [12] and is a useful predictor of decreased gait speed [8]. On the other hand, spatiotemporal gait parameters other than gait speed, such as step length, were also reported to be associated with fall-related self-efficacy [13–15]. Thus, it is strongly assumed that fall-related self-efficacy and gait function in older people are associated with each other. However, whether the interaction between fall-related self-efficacy and gait function affects future falls has not been clarified. Thus, the aim of this study was to verify associations between fall-related self-efficacy and spatiotemporal gait parameters, and to investigate the effect of the interaction between fall-related self-efficacy and gait function on the occurrence of falls in community-dwelling older people.

## Methods

### Subjects

The participants of the present study were elderly persons (age ≥ 65 years) who lived in Sagamihara City, Kanagawa prefecture, Japan. They were recruited from August 2017 to February 2018 via advertisements in newspapers and community newsletters. The inclusion criteria of this study were the following: (1) age ≥ 65 years at the time of data collection; (2) living in the community; and (3) independent in activities of daily living (ADL). Independency of ADL was checked by the presence or absence of certification for long-term care insurance in Japan. The exclusion criteria were: (1) living in facilities such as a nursing care home; (2) judged as having a care level for certification for long-term care insurance in Japan; (3) severe cardio-pulmonary disease and neurological disease; and (4) limitations preventing them from participating in the gait and physical function tests described below. The inclusion and exclusion criteria were checked by trained researchers via questionnaires and interviews before data collection. A total of 265 older people were eligible, and all data collection was performed at the same public recreation facility in Sagamihara City.

The present study was approved by the Institutional Review Board of the School of Allied Health Sciences at Kitasato University (approval number 2018-008B), and written, informed consent was obtained from all participants.

### Gait function: measurements of spatiotemporal gait parameters

As an assessment of gait function, spatiotemporal gait parameters were measured using a mat-type walkway that was 2.4 m in length and 60 cm in width with embedded pressure sensors (WalkWay, MW-1000; ANIMA Corp., Tokyo, Japan). Acceleration and deceleration zones (each 3.3 m) were set at the start and end of the walkway system, and the participants were instructed to walk from the edge of the acceleration zone to the end of the deceleration zone (9 m in total) at their usual pace and at their maximum effort pace. The measurements were performed two times each at the usual pace and at the maximum pace. The methods and validity of the measurements of gait function in this study have been described in detail elsewhere [16]. As spatiotemporal gait parameters, speed (cm/sec), stride time (sec), step length (cm), step width (cm), cadence (steps/min), stance time (% stride time), and double support time (% stride time) were collected by the walkway system. The definition of each spatiotemporal gait parameter corresponded to that in a previous study [17]. For all spatiotemporal gait parameters, the mean values of the two measurements at usual and at maximum paces were used for statistical analysis. Furthermore, the changes in each spatiotemporal gait parameter between usual and maximum paces (Δgait parameter) were also calculated as an assessment of gait function in the present study (Δgait parameter = gait parameter at maximum pace—gait parameter at usual pace).

### Fall-related self-efficacy

Fall-related self-efficacy was assessed using the short version of the Falls Efficacy Scale International (Short FES-I) [18]. The Short FES-I has been translated into a Japanese version, whose reliability and validity have been confirmed [19]. The Short FES-I is a self-rated questionnaire consisting of 7 items, and each item is rated using a 4-point Likert scale (minimum of 7 points, maximum of 28 points). A higher point score indicates lower self-efficacy, and the cutoff for a high risk of falls in Japanese older people was reported to be 13 points [8]. Thus, the result of the Short FES-I was categorized into low efficacy and high efficacy based on the cutoff point (< 13 points for high efficacy, ≥ 13 points for low efficacy) for further analysis. All participants were blinded to the results of individuals’ efficacy levels (low or high), because that information could bias the results of this study.

### Occurrence of falls

The occurrence of falls was prospectively investigated by questionnaire and face-to-face interview 6 months after the measurements of gait parameters and the Short FES-I. The definition of falls in this study referred to a previous study [20]. During the 6-month follow-up period, those who reported one or more falls were defined as fallers, and those who reported no falls were defined as non-fallers.

### Confounding factors

Body mass index (BMI), physical functions, depressive symptoms, instrumental ADL (IADL), medications, and fall history were collected as confounding factors. BMI was calculated from weight and height. As physical functions, grip strength and the Timed Up and Go (TUG) test [21] were measured. The measurement methods for grip strength and the TUG test have been described in detail elsewhere [19]. Depressive symptoms were assessed using the 5-item geriatric depression scale (5-GDS), and the presence of absence of depressive symptoms was judged by the cutoff point determined in a previous study [22]. IADL was assessed using a subscale of the Tokyo Metropolitan Institute of Gerontology Index of Competence (TMIG-IC) [23], and a full score (5 points) on the IADL scale of the TMIG-IC was defined as independent in IADL. In addition, the number of prescribed medications and the presence or absence of two or more falls within 6 months were also evaluated.

### Statistical analysis

For verification of associations between fall-related self-efficacy and gait function, the differences between high efficacy and low efficacy in all gait parameters were analyzed using unpaired *t* tests. Then, the differences between fallers and non-fallers in all gait parameters, fall-related self-efficacy (high and low efficacy), and confounding factors were analyzed using the chi-squared test or the unpaired *t* test. For fall-related self-efficacy and the gait parameters that were found to show significant differences between fallers and non-fallers, multivariate logistic regression analysis adjusted for potential confounding factors was performed, with the occurrence of falls (fallers and non-fallers) set as the dependent variable and fall-related self-efficacy or the gait parameters set as the independent variables. Finally, to investigate the effect of the interaction between fall-related self-efficacy and gait function on the occurrence of falls, multivariate logistic regression analysis adjusted for potential confounding factors was performed, with the occurrence of falls set as the dependent variable, and the interaction term of fall-related self-efficacy and gait parameters set as the independent variable. With respect to the interaction term, the gait parameters that showed significant differences between the low-efficacy and high-efficacy groups were identified as the variables combined with fall-related self-efficacy. All statistical analyses were performed using the R programming language and environment (R version 3.2.2) [24], with the level of significance set at 5%.

## Results

The basic attributes of the 265 participants are shown in Table 1. Of the 265 participants, 204 (77.0%) participated in the follow-up survey on the occurrence of falls after 6 months. Basic attributes, all gait parameters except for stance time and double support time at maximum pace, and fall-related self-efficacy were not significantly different between the follow-up participants and the non-follow-up participants.

**Table 1:** Basic attributes of the participants

	All participants
	*n* = 265
	Mean (SD)/Number (%)
Age (years)	72.9 (5.1)
Sex (woman)	165 (62.3)
Body mass index (kg/m^2^)	22.1 (2.9)
Short FES-I^a^ (/28 points)	11.4 (3.6)
Grip (kg)	27.6 (7.3)
Timed Up and Go test (s)	6.0 (1.0)
Two or more falls in the previous 6 months	6 (2.3)
IADL^b^ (independent)	238 (89.8)
Depressive symptoms	36 (13.6)
Number of medications (types/days)	1.1 (1.0)

^a^*Short FES-I* short version of the Falls Efficacy Scale International

^b^*IADL* instrumental activities of daily living

As for fall-related self-efficacy, 100 people (37.7%) were categorized into the low-efficacy group. As for the spatiotemporal gait parameters, the low-efficacy group had significantly slower speed and shorter step length at maximum pace than the high-efficacy group (Table 2). Of the Δgait parameters, Δspeed, Δstance time, and Δdouble support time were significantly smaller in the low-efficacy group than in the high-efficacy group (Table 2).

**Table 2:** Comparison of gait function between high and low fall-related self-efficacy groups

	Low-efficacy group	High-efficacy group	*p* value
Gait function	*n* = 100	*n* = 165	
	Mean (SD)/Number (%)		
Gait parameters at usual pace			
Speed (cm/s)	146.8 (22.6)	148.4 (23.1)	0.595
Step length (cm)	66.5 (8.6)	67.4 (8.0)	0.393
Step width (cm)	8.3 (2.5)	8.2 (2.7)	0.813
Cadence (steps/min)	133.2 (15.5)	131.9 (11.3)	0.456
Stride time (s)	0.9 (0.09)	0.9 (0.08)	0.855
Stance time (% stride time)	59.0 (1.8)	59.1 (1.8)	0.808
Double support time (% stride time)	9.7 (1.6)	9.8 (1.5)	0.846
Gait parameters at maximum pace			
Speed (cm/s)	187.5 (29.2)	195.9 (31.2)	0.030
Step length (cm)	72.8 (9.1)	75.2 (8.8)	0.037
Step width (cm)	7.5 (2.5)	7.9 (3.2)	0.276
Cadence (steps/min)	154.0 (18.3)	156.5 (18.2)	0.290
Stride time (s)	0.8 (0.09)	0.8 (0.09)	0.305
Stance time (% stride time)	56.9 (2.2)	57.4 (2.3)	0.091
Double support time (% stride time)	8.3 (2.0)	7.9 (2.0)	0.094
Δgait parameters			
Δspeed (cm/s)	40.8 (21.0)	47.5 (24.3)	0.026
Δstep length (cm)	6.3 (6.8)	7.8 (6.6)	0.069
Δstep width (cm)	− 0.8 (2.3)	− 0.3 (2.4)	0.101
Δcadence (steps/min)	21.5 (16.7)	24.5 (16.5)	0.171
Δstride time (sec)	− 0.1 (0.1)	− 0.1 (0.1)	0.387
Δstance time (% stride time)	− 1.7 (2.1)	− 2.2 (2.1)	0.047
Δdouble support time (% stride time)	− 1.5 (1.5)	− 1.9 (1.5)	0.033

Low efficacy: Short FES-I ≥ 13 points, High efficacy: Short FES-I < 13 points

Δgait parameters = (gait parameters at maximum pace—gait parameters at usual pace)

At the follow-up survey, 25 people (12.3%) reported one or more falls during the 6 months that had passed. The basic attributes of the fallers and non-fallers are shown in Table 3. With respect to fall-related self-efficacy, significantly more fallers had low self-efficacy than non-fallers. Even after adjustment for confounding factors, low self-efficacy was significantly associated with falls (Table 4). Of the gait parameters, only Δdouble support time was significantly different between fallers and non-fallers; the change in double support time was smaller in fallers than in non-fallers. Similar to fall-related self-efficacy, even after adjustment for confounding factors, Δdouble support time was significantly associated with falls (Table 4).

**Table 3:** Comparison of basic attributes of the participants between non-fallers and fallers during 6-month follow-up

	Falls during 6 months		*p* value
	Non-fallers	Fallers	
	*n* = 179	*n* = 25	
	Mean (SD) or Number (%)		
Age (years)	73.0 (5.1)	74.2 (5.7)	0.277
Sex (woman)	112 (62.6%)	18 (72.0%)	0.486
Body mass index (kg/m^2^)	22.2 (2.7)	21.2 (2.7)	0.082
Grip (kg)	27.6 (7.3)	25.0 (5.7)	0.093
Timed Up and Go test (s)	6.0 (1.0)	6.3 (1.3)	0.221
Two or more falls in the previous 6 months	1 (0.6%)	4 (16.0%)	< 0.001
IADL^a^ (independent)	165 (92.2%)	22 (88.0%)	0.748
Depressive symptoms	19 (10.7%)	8 (32.0%)	0.009
Number of medications (types/days)	1.1 (1.0)	1.3 (0.9)	0.257

^a^*IADL* instrumental activities of daily living

**Table 4:** Occurrence of falls and fall-related self-efficacy, gait function, and the interaction between fall-related self-efficacy and gait function

	Faller during 6-month follow-up	
	OR (95% CI)	*p* value
Fall-related self-efficacy and gait function		
Low efficacy (vs. high efficacy)	2.72 (1.05:7.06)	0.039
Δdouble support time (per 1%)	1.04 (1.00:1.07)	0.039
Interaction between self-efficacy and speed		
Low efficacy * Speed (per 1 cm/s)	1.01 (0.99:1.02)	0.427
High efficacy * Speed (per 1 cm/s)	1.00 (0.99:1.02)	0.834
Interaction between self-efficacy and step length		
Low efficacy * Step length (per 1 cm)	1.05 (0.99:1.12)	0.127
High efficacy * Step length (per 1 cm)	1.04 (0.97:1.11)	0.255
Interaction between self-efficacy and Δspeed		
Low efficacy * Δspeed (per 1 cm/s)	1.01 (0.99:1.04)	0.391
High efficacy * Δspeed (per 1 cm/s)	0.99 (0.97:1.02)	0.457
Interaction between self-efficacy and Δstance time		
Low efficacy * Δstance time (per 1%)	0.99 (0.73:1.34)	0.923
High efficacy * Δstance time (per 1%)	1.59 (1.05:2.39)	0.027
Interaction between self-efficacy and Δ double support time		
Low efficacy * Δdouble support time (per 1%)	1.17 (0.76:1.80)	0.478
High efficacy * Δdouble support time (per 1%)	1.67 (1.04:2.71)	0.035

The odds ratios were adjusted for age, sex, BMI, fall history, depressive symptoms, and grip strength. Low efficacy was defined as those who had ≥ 13 points on the short FES-I, and high efficacy was defined as < 13 points. The Δstance time and Δdouble support time were calculated by subtracting stance time and double support time at the usual pace from that at the maximum pace

**Fig. 1:**
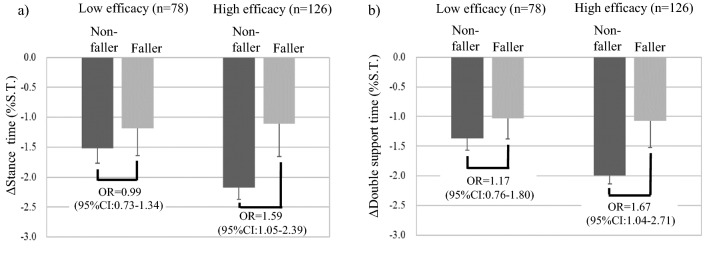
Effect of the interaction between fall-related self-efficacy and gait function on the occurrence of falls. **a** Δstance time, **b** Δdouble support time. S.T. on the vertical axis is stride time. The odds ratio in the figure was adjusted by age, sex, BMI, fall history, depressive symptoms, and grip strength

As for the interactions between fall-related self-efficacy and gait function, the interaction term of fall-related self-efficacy and Δstance time and the interaction term of fall-related self-efficacy and Δdouble support time were significantly associated with the occurrence of falls. Namely, smaller Δstance time or Δdouble support time in those with high efficacy was associated with a higher odds ratio (OR) of falls, whereas Δstance time and Δdouble support time in those with low efficacy were not associated with falls (Table 4, Fig. 1).

## Discussion

In the present study, the associations between fall-related self-efficacy and speed and step length in gait parameters at maximum pace were clarified. These findings agreed with those of previous studies [12–15]; therefore, fall-related self-efficacy appears to be definitely associated with spatiotemporal gait parameters and to be an index reflecting not only gait speed, but also gait patterns for older people. In the present study, the changes in gait parameters between usual and maximum paces (Δgait parameters) were also related to fall-related self-efficacy. In a previous study, the difference in gait speed between usual and maximum paces was defined as walking speed reserve, and a smaller walking speed reserve was reported to be associated with cognitive decline in older people [25]. Similarly, Δgait parameters as reported in the present study appear to indicate so-called gait function reserve in older people; thus, decreased gait function reserve may lead to a decline of fall-related self-efficacy. However, the clinical meanings of the Δgait parameters or gait function reserve need to be investigated in future studies.

As described above, fall-related self-efficacy and gait function were shown to have a definite association. On the other hand, the results of the present study also indicated that fall-related self-efficacy and Δdouble support time were each independent risk factors for falls. Fall-related self-efficacy has already been shown to be associated with future falls in previous studies [5–8]; the result of the present study agreed with the results of those studies. With respect to gait function, gait speed has been found to be a predictor of future falls [3, 4]. Furthermore, in addition to gait speed, it has been suggested that step length, double support time, and gait variability are associated with the occurrence of falls [4, 13, 26]. Therefore, because various gait parameters reflect on gait function and can be associated with fall risk factors, the finding that one of the Δgait parameters that may reflect gait function reserve was significantly associated with falls was not unexpected.

However, few studies have investigated the effect of the interaction of fall-related self-efficacy and gait function on the occurrence of falls. In the present study, the interaction between fall-related self-efficacy and Δgait parameters was associated with future falls, even after adjustment for confounding factors. Thus, not only fall-related self-efficacy and gait function were each independent fall risk factors, but also the interaction between both factors was found to affect future falls. In a previous study, a discrepancy between self-reported subjective physical function and test-based objective physical function was reported to be associated with future falls [27]. Furthermore, a discrepancy between self-reported mobility and test-based mobility was reported to be associated with mortality [28]. In addition, the interaction between subjective cognition and objective cognition was also found to have an effect on actual memory performance in previous studies [29]. From the result of the present study, older people with a smaller Δdouble support time or Δstance time despite high self-efficacy tended to have a risk of future falls. Therefore, even if self-efficacy, which is a subjective assessment, is maintained at a high level, when gait function or gait function reserve, which is an objective assessment, is in decline, fall risk may be increased.

For prediction of future falls, spatiotemporal gait parameters and performance tests such as the TUG test, which are objective assessments of physical factors, have been shown to have insufficient predictive accuracy [30–32]. Similarly, the predictive accuracy of a scale for fall-related self-efficacy, which is a subjective assessment of psychological factors, was also suggested to be insufficient [8]. The findings of the present study suggest that the accuracy of identification of older people with a high risk for falls may be improved by combined assessment of subjective/psychological risk factors such as fall-related self-efficacy and objective/physical risk factors such as gait function. However, in order to determine an algorithm and an accurate fall risk assessment considering the interaction between subjective/psychological and objective/physical factors, further studies are needed.

In this study, fall-related self-efficacy was evaluated as a psychological factor related to fall risk in older people. On the other hand, it has also been suggested that cognitive function, such as executive function, as one of the psychological factors, is associated with falls [33]. As for the relationship between cognitive function and falls, older people with the combination of slow gait speed and mild cognitive impairment (MCI) were reported to have a higher fall risk than those with only slow gait speed or only MCI [34]. Therefore, a combined assessment of cognitive function and gait function may be useful to predict falls. However, evaluation of cognitive function of older people is not easy and simple from the point of feasibility as compared to fall-related self-efficacy. In fact, testing cognitive functions in multiple domains, such as memory, attention, and language is necessary to assess MCI. However, the short FES-I used in this study can be assessed by just 7 questionnaire items; thus, use of this scale in local community and clinical settings is a strength.

The present study had several limitations. All of the participants of this study were independent in ADL, and about 90% were also independent in IADL. Thus, since almost all of the participants maintained good functional status, the effect of bias of the participants’ attributes on the results cannot be completely ruled out. Furthermore, about 20% of the participants could not complete the follow-up survey on the occurrence of falls. The bias caused by the missing data was estimated to be slight, but one cannot say that the missing data had absolutely no effect on the results of this study. Finally, it has been suggested that fall-related self-efficacy is affected by cultural differences [35]. Therefore, when the results of this study are generalized to older populations other than Japanese, they must be interpreted carefully.

In conclusion, fall-related self-efficacy and spatiotemporal gait functions were associated with each other, and they were each independent risk factors for falls. Furthermore, the interaction between fall-related self-efficacy and gait function appeared to affect fall risk. Assessments that include fall-related self-efficacy and gait function may improve the accuracy of predicting the occurrence of falls.

## Data Availability

The datasets generated and analyzed during the current study are available from the corresponding author on reasonable request.
